# Association between gut microbiota and autoimmune cholestatic liver disease, a Mendelian randomization study

**DOI:** 10.3389/fmicb.2024.1348027

**Published:** 2024-03-27

**Authors:** YangLin Cui, YuMeng Guo, YuChen Kong, GuangYe Zhang

**Affiliations:** ^1^First Clinical College of Medicine, Shandong University of Chinese Medicine, Jinan, China; ^2^Classical Chinese Medicine Section, Rizhao Hospital of Traditional Chinese Medicine, Rizhao, China

**Keywords:** gut microbiota, autoimmune cholestatic liver disease, Mendelian randomization, primary biliary cholangitis, primary sclerosing cholangitis

## Abstract

**Background:**

Previous studies have suggested that the gut microbiota (GM) is closely associated with the development of autoimmune cholestatic liver disease (ACLD), but limitations, such as the presence of confounding factors, have resulted in a causal relationship between the gut microbiota and autoimmune cholestatic liver disease that remains uncertain. Thus, we used two-sample Mendelian randomization as a research method to explore the causal relationship between the two.

**Methods:**

Pooled statistics of gut microbiota from a meta-analysis of genome-wide association studies conducted by the MiBioGen consortium were used as an instrumental variable for exposure factors. The Pooled statistics for primary biliary cholangitis (PBC) and primary sclerosing cholangitis (PSC) were obtained from the R9 version of the FinnGen database (https://r9.finngen.fi/). Inverse-variance Weighted (IVW), cML-MA, MR-Egger regression, Weighted median (WME), Weighted mode (WM), and Simple mode (SM) were used to detect the association between intestinal flora and the causal relationship between intestinal flora and ACLD, in which IVW method was dominant, was assessed based on the effect indicator dominance ratio (odds ratio, OR) and 95% confidence interval (CI). Sensitivity analysis, heterogeneity test, gene pleiotropy test, MR pleiotropy residual sum and outlier test (MR-PRESSO) were combined to verify the stability and reliability of the results. Reverse Mendelian randomization analysis was performed on gut microbiota and found to be causally associated with ACLD.

**Results:**

The IVW results showed that the relative abundance of the genus *Clostridium innocuum* group, genus *Butyricicoccus*, and genus *Erysipelatoclostridium* was negatively correlated with the risk of PBC, that is, increased abundance reduced the risk of PBC and was a protective, and the relative abundance of the genus *Eubacterium hallii* was positively correlated with the risk of PSC, which is a risk factor for PSC. Family *Clostridiaceae1* and family *Lachnospiraceae* were negatively correlated with the risk of PSC, which is a protective factor for PSC.

**Conclusion:**

This study found a causal relationship between gut microbiota and ACLD. This may provide valuable insights into gut microbiota-mediated pathogenesis of ACLD. It is necessary to conduct a large-sample randomized controlled trial (RCT) at a later stage to validate the associated role of the relevant gut microbiota in the risk of ACLD development and to explore the associated mechanisms.

## 1 Introduction

Autoimmune cholestatic liver disease (ACLD) encompasses a cadre of immune-mediated, protracted cholestatic hepatic maladies, notably primary biliary cholangitis (PBC) and primary sclerosing cholangitis (PSC). The etiology of ACLD is the complicated interplay between genetic predispositions and environmental factors, precipitating the breakdown of immune tolerance toward biliary epithelial cells and subsequent immune activation (Liwinski et al., 2022; Richardson et al., 2022). Among these, PBC is characterized by progressive destruction of the small intrahepatic bile ducts as a pathological feature, whereas PSC manifests inflammation or fibrosis within both intrahepatic and extrahepatic conduits of medium and large calibers (Blesl and Stadlbauer, 2021). The pathophysiological mechanisms underlying ACLD remain unclear. In recent years, scholarly attention has pivoted toward the gut microbiota (GM) as a critical environmental determinant, with multiple investigations revealing a potential correlation between GM and ACLD. For instance, Furukawa et al. (2020) observed a decrease in the microbial diversity of patients with PBC, characterized by an elevated relative abundance of *Lactobacilli* and a diminished presence of symbiotically beneficial *Clostridium* compared to their normal counterparts. Moreover, evidence suggests that GM and its metabolites may be intricately linked to the progression of PBC toward hepatic fibrosis (Lammert et al., 2021). Analogously, investigations into PSC have revealed reduced GM diversity and altered abundance of specific flora. Fukui (2019), in an observational study, identified an upregulation of fecal *Haemophilus*, *Rothia*, *Clostridium*, *Enterococcus*, *Streptococcus*, and *Veillonella* in 43 Czech PSC patients compared to their healthy counterparts. Notwithstanding the aforementioned insights, observational studies encounter the challenge of controlling for confounding variables such as age, environment, dietary habits, and lifestyle, which may impinge upon the discernment of a causal relationship between GM and ACLD. Consequently, to clarify the potential causal nexus between GM and ACLD, we deployed a two-way Mendelian randomization approach.

Mendelian randomization (MR) is a methodological approach that leverages genetic variation to construct instrumental variables (IVs) for exposure, thereby facilitating the estimation of causal relationships between said exposure and the onset of a given malady (Greenland, 2000). It is a viable surrogate for Randomized Controlled Trials (RCTs), owing to the randomized assignment of genotypes from progenitors to progeny (Zhang et al., 2023). This randomization process ensures that the association between genetic variants and the resultant outcome remains impervious to common confounding variables, and the causal sequence maintains a plausible trajectory (Li et al., 2022). The methodological prowess of MR is now ubiquitously harnessed to dig deeper into the complicated tapestry of causal relationships between the intricacies of the GM and afflictions. In this study, we meticulously employed Genome-Wide Association Study (GWAS) amalgamated data from MiBioGen and the R9 version of the FinnGen Consortium to perform a two-sample Mendelian randomization (TSMR) analysis to assess the causal relationship between GM and ACLD.

## 2 Materials and methods

### 2.1 Study design

In the present inquiry, we applied TSMR methodology to scrutinize the complicated nexus between GM and ACLD. This investigation involved orchestration of dual, bidirectional, two-sample Mendelian randomization analyses, employing GM as the exposure, PBC and PSC as the outcomes. Our primary analytical approach predominantly involved the Inverse Variance Weighting (IVW) method, with subsequent layers of scrutiny including sensitivity analyses, heterogeneity assessments, gene multiplicity evaluations, and application of the MR-PRESSO algorithm, all orchestrated to corroborate the steadfast reliability of the derived outcomes.

**Figure 1:**
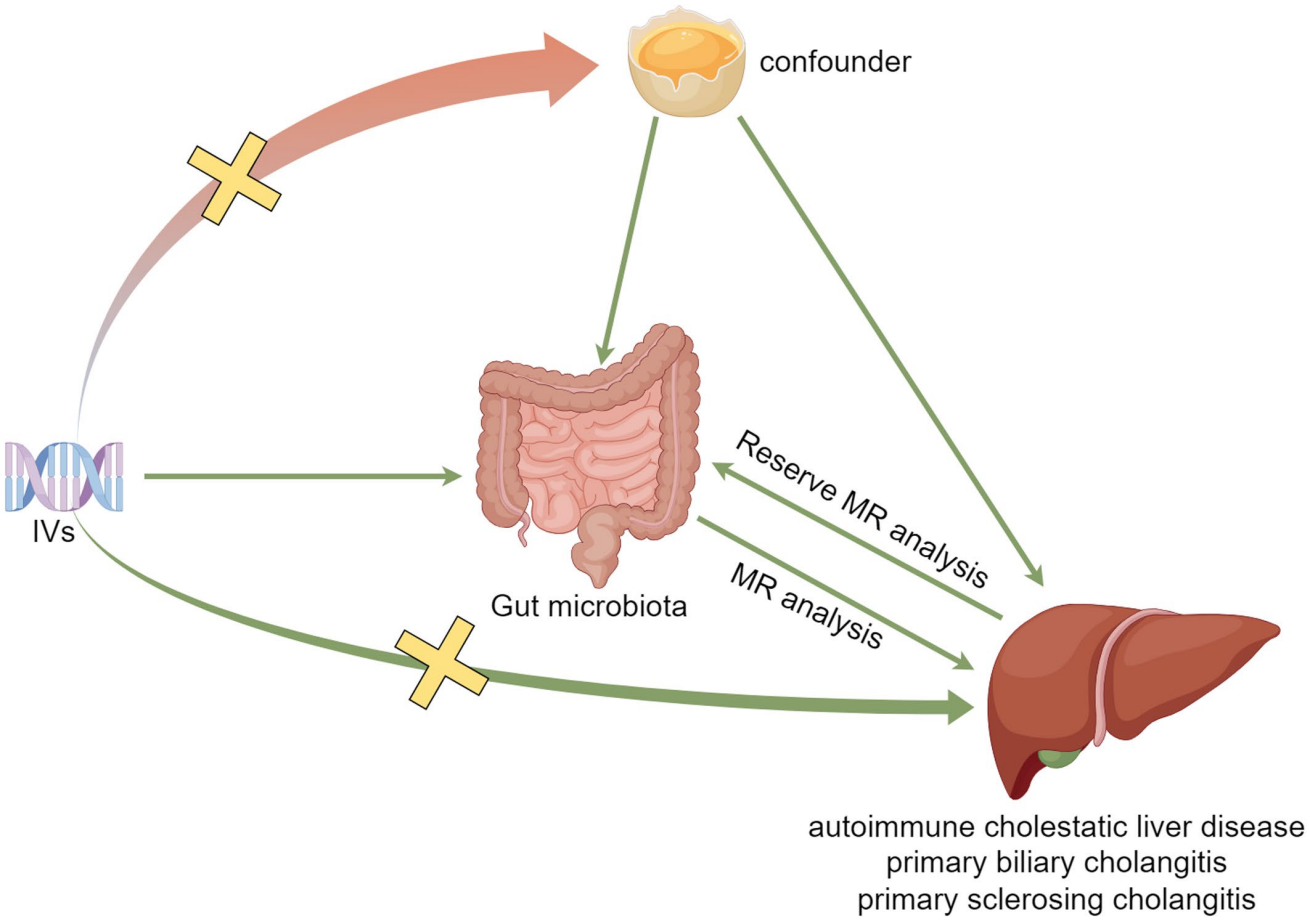
Model plot for MR analysis between GM and ACLD.

MR analysis needs to satisfy the following three assumptions: (1) IVs and exposure need to be strongly associated (association assumption), (2) IVs should not be associated with the outcome through confounders (independence assumption), and (3) IVs cannot directly affect the outcome but only through exposure (exclusivity assumption). The specific process is shown in Figure 1.

### 2.2 Data sources

Single nucleotide polymorphisms (SNPs) intricately linked to the gut microbiota (GM) have been meticulously curated as instrumental variables for exposure. The consolidated dataset for these SNPs emanated from the MiBioGen consortium, an august assembly that scrutinized a comprehensive assemblage of 18,340 samples featuring 16S rRNA gene sequencing, drawn from a mosaic of 24 population-based cohorts, predominantly of European lineage. The microbial composition was meticulously analyzed, targeting three distinct variable regions of the 16S rRNA gene (V4, V3-V4, and V1-V2), with subsequent taxonomic classification achieved through direct taxonomic binning. To discern the complicated nuances of host genetic influence on gut bacterial taxa abundance, the endeavor embraced Microbiota Quantitative Trait Locus (mbQTL) positional analyses (Kurilshikov et al., 2021). Genetic datasets pertinent to both PBC and PSC—integral components of ACLD spectrum—were gleaned from the R9 iteration of the FinnGen Consortium, accessible at https://r9.finngen.fi/ (Kurki et al., 2023). The PBC cohort comprised 281,684 participants, with the case group numbered 557, and the healthy control cohort comprised 281,127 individuals. This collective was characterized by an exhaustive 20,167,312 SNPs. In parallel, the PSC dataset encompasses 332,618 participants, including 1,715 in the case group and 330,903 in the healthy control cohort, with an analogous wealth of 20,169,171 SNPs. Detailed information on ACLD data can be found in Supplementary Table 1.

### 2.3 Selection of instrumental variables

A meticulous screening process was undertaken to identify IVs from a cohort of 211 gut microbes, adhering to the following exclusion criteria: (1) SNPs achieving a significance threshold (*p* < 1.0 × 10^−5^) within the designated locus range were discerningly chosen as potential IVs (Zhang et al., 2023). (2) To guarantee the independence of the included SNPs, linkage disequilibrium (LD) was meticulously expunged using a reference panel sourced from 1,000 Genome Project European samples, with a stringent criterion of *r*^2^ < 0.001 and a window size of 10,000 kb. Finally, data for the above selected SNPs were extracted from the outcome variables.

To obviate the potential sway of feeble instrumental biases in the estimation of the association effect, a rigorous evaluation of the robustness of instrumental variables was conducted. This scrutiny involved a meticulous assessment of the *F*-value, derived from the formula *F* = β^2^/se^2^ (Xie et al., 2023), where β represents the effect value and se signifies the standard error. It was posited that the absence of significant weak instrumental bias could be affirmed when the calculated *F*-value exceeded the threshold of 10 (Staiger and Stock, 1997).

### 2.4 Statistical methods

Within the confines of this investigation, the primary modality employed to scrutinize the potential causal linkage between gut microbial abundance and the proclivity for ACLD rested on IVW analysis. This was seamlessly complemented by an ensemble of supplementary methodologies, including cML-MA, MR-Egger, Weighted median (WME), Weighted mode (WM), and Simple mode (SM). The imprimatur of a relatively steadfast causal association was imputed when the *p*-value fell below 0.05 for the IVW methods and concordantly for cML-MA, MR-Egger, WME, WM, and SM when their vectors were aligned (Zhang et al., 2023). Furthermore, a meticulous dissection of the MR-Egger intercept term, denoted as the Egger-intercept, served as a sentinel for horizontal pleiotropy. An Egger-intercept infinitesimally proximal to 0 or statistically insignificant signaled the dearth of genetic pleiotropy (Burgess and Thompson, 2017). Additionally, the study used the MR-PRESSO methodology, an instrument adept at identifying potential outliers. Subsequent to the excision of outliers (*p*-value <0.05), a recalibration of the causal analysis was performed (Verbanck et al., 2018). Sensitivity analyses, executed using the leave-one-out method, were instrumental in gauging the impact of each SNP on causality. This intricate procedure involves the sequential removal of individual SNPs to ascertain the cumulative effect value of the residual SNPs (Hemani et al., 2018). To gauge the specter of heterogeneity, the study invoked the Cochran’s *Q* test was used to discern potential discordance among the instrumental variables. A *p*-value less than 0.05 signified a noteworthy presence of heterogeneity. In a judicious effort to avert spurious positives due to the vicissitudes of multiple testing, a stringent false discovery rate (FDR) correction was implemented. Results attaining a *P*FDR < 0.1 were deemed statistically significant (Storey and Tibshirani, 2003; Li et al., 2022). In a bid to unravel the causal intricacies between GM and ACLD, a reciprocal MR analysis was executed for bacterial taxa pinpointed as causally entwined with ACLD in antecedent MR exploration. The genome-wide significance threshold for the exposure dataset was rigidly set at *p* < 5.0 × 10^−6^, with all other criteria and parameters aligned harmoniously with those governing anterior MR scrutiny.

Statistical analyses were diligently executed employing the Two Sample MR package (version 0.5.7) (Hemani et al., 2018), the MR-PRESSO package (version 1.0) (Verbanck et al., 2018), and MR-cML (Xue et al., 2021) within the expanse of the R software (version 4.3.1).

## 3 Results

### 3.1 Instrumental variable

Eradicating 15 unclassified genera, a collective of 196 intestinal microorganisms spanning 9 phyla, 16 orders, 20 orders, 32 families, and 119 genera were judiciously included in this inquiry. Following the stringent criteria delineated in the previous section, 2,774 SNPs (Supplementary Table 2) intricately linked to the intestinal flora were identified. Notably, each of these SNPs boasted *F*-values surpassing the threshold of 10, thus effectually obviating the distortions introduced by feeble instrumental variables.

### 3.2 Mendelian randomization analysis

**Figure 2:**
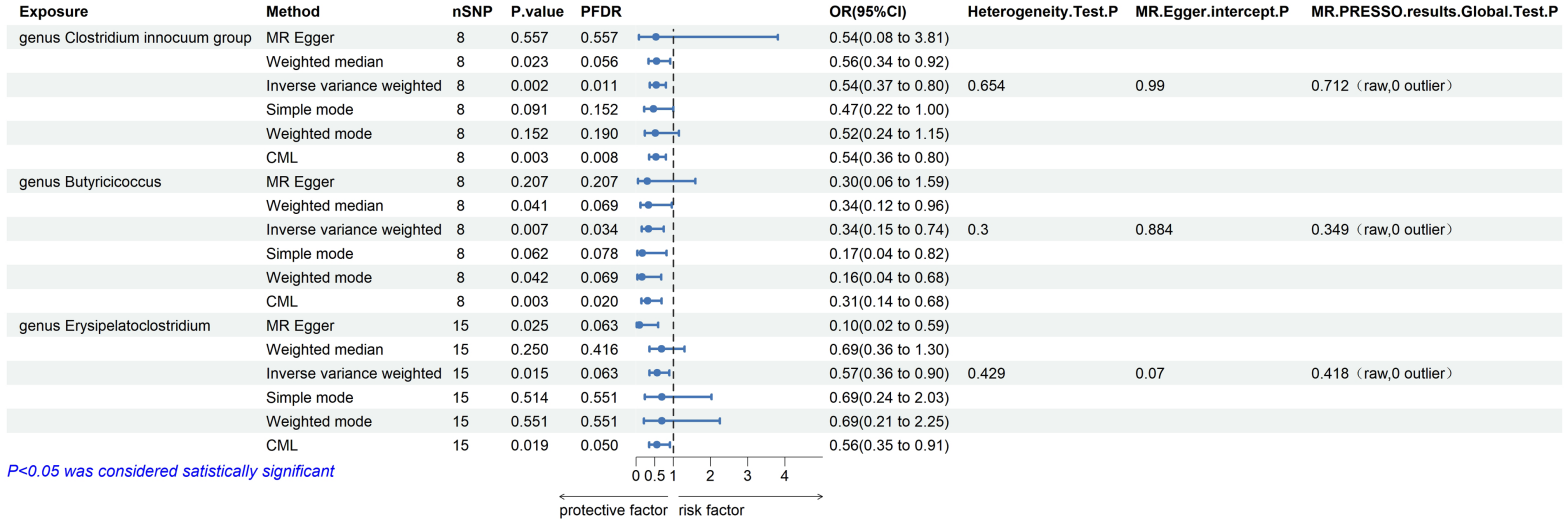
Results of MR analysis of 6 methods of GM and PBC and Egger-intercept, MR-PRESSO, and Cochran Q tests of IVW methods.

**Figure 3:**
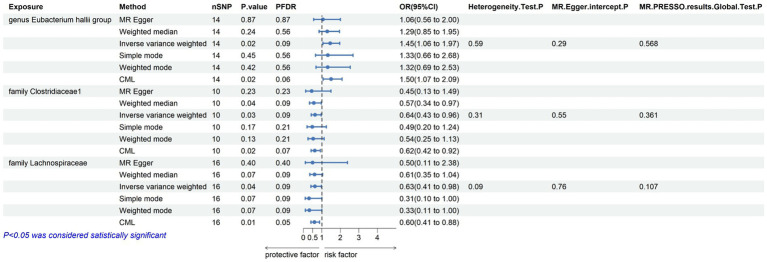
Results of MR analysis of 6 methods of GM and PSC and Egger-intercept, MR-PRESSO, and Cochran Q tests of IVW methods.

**Figure 4:**
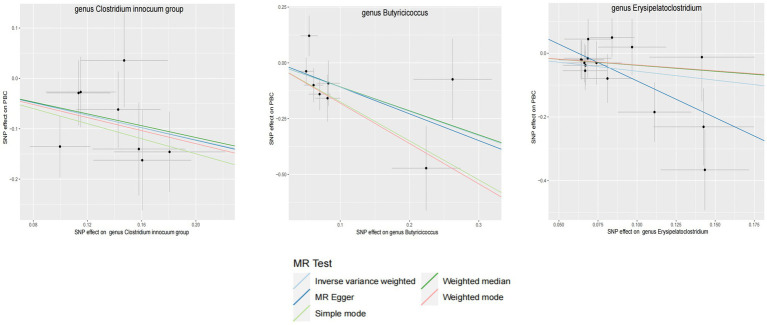
GM with causal relationship with PBC and the scatterplot of the 6 MR models.

**Figure 5:**
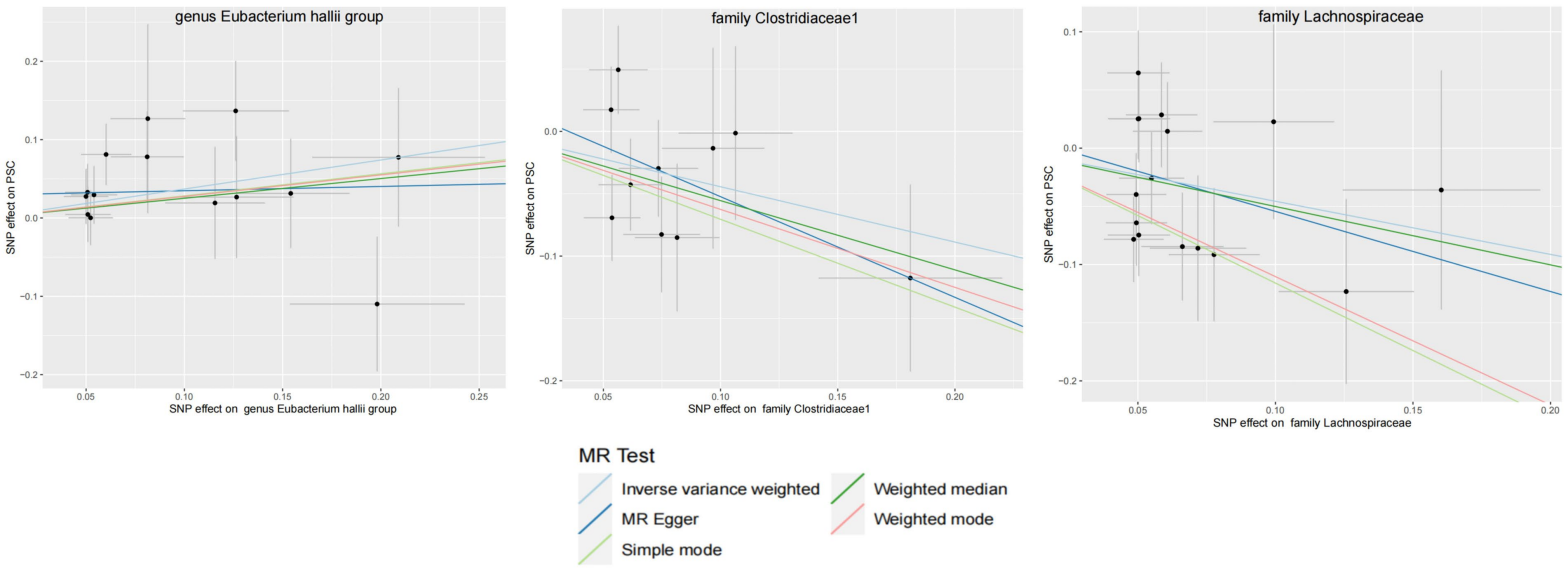
GM with causal relationship with PSC and the scatterplot of the 6 MR models.

Within this investigation, a compendium of six MR methodologies was meticulously employed, with subsequent result scrutiny via the FDR correction method. In particular, Inverse-variance Weighted (IVW) outcomes unveiled six discernible gut microorganisms intricately implicated in the etiology of ACLD, each registering a noteworthy statistical significance (*P*FDR < 0.1). The relative abundance of three genera, including genus *Clostridium innocuum* group (OR = 0.54, 95% CI: 0.37–0.80, PFDR = 0.011), genus *Butyricoccus* (OR = 0.34, 95% CI: 0.15–0.74, *P*FDR = 0.034), and genus *Erysipelatoclostridium* (OR = 0.57, 95% CI: 0.36–0.90, *P*FDR = 0.063), and the relative abundance of the three types of intestinal microorganisms were negatively correlated with the risk of developing PBC, and the above three types of intestinal microorganisms were protective factors for PBC; the relative abundance of genus *Eubacterium hallii* group (OR = 1.45, 95% CI: 1.06–1.97, *P*FDR = 0.09) was positively correlated with the risk of developing PSC, i.e., it was a risk factor for PSC, family *Clostridiaceae1* (OR = 0.64, 95% CI: 0.43–0.96, *P*FDR = 0.09) and family *Lachnospiraceae* (OR = 0.63, 95% CI: 0.41–0.98, *P*FDR = 0.09) were negatively correlated with the incidence of PSC, and were protective factors for PSC thereby manifesting as protective factors. In tandem, the complementary method cML-MA-BIC lent credence to the causal associations discerned in the aforementioned six gut microbial contributions to ACLDs. Visual representations in Figures 2–5 concurred with the directional alignment of effect values across the six Two-Sample Mendelian Randomization (TSMR) methodologies, solidifying the inference that the six identified intestinal bacterial taxa were substantively and causally interwoven with the complex etiology of ACLD, thereby accentuating the robustness of the results.

### 3.3 Quality control

**Figure 6:**
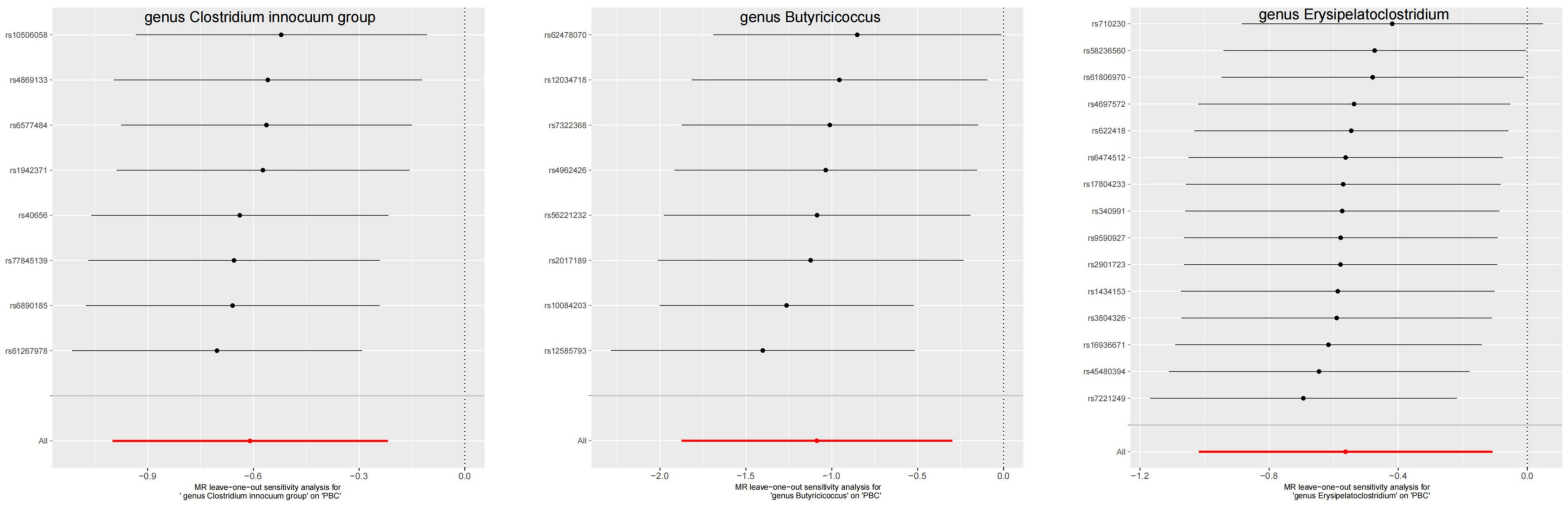
The leave-one-out plots of GM with causal relationship with PBC.

**Figure 7:**
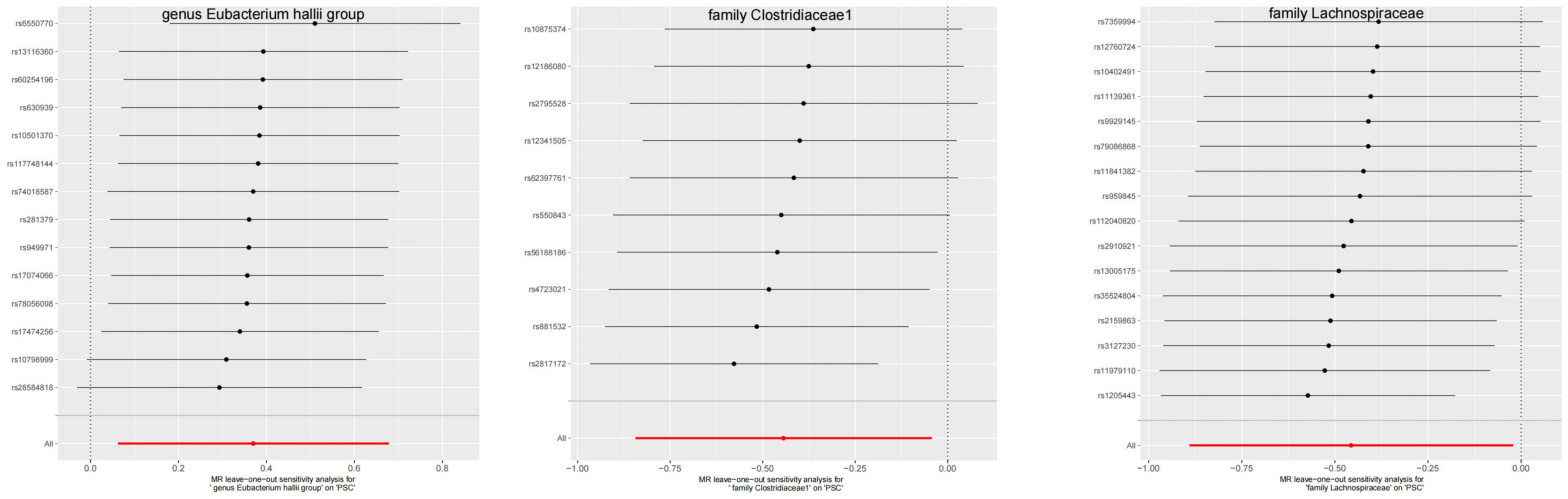
The leave-one-out plots of GM with causal relationship with PSC.

The leave-one-out method analysis meticulously demonstrated that the stepwise exclusion of SNPs engendered no substantive deviation between the amalgamated effect and cumulative effect of the residual SNPs, as elegantly delineated in Figures 6, 7. Akin to this, the discerning MR-PRESSO analysis, expounded in Figures 2, 3, corroborated the absence of outliers (*p* > 0.05), thereby amplifying the veracity of the results. Furthermore, both the Cochran Q test and the Egger-intercept test unveiled *p* values surpassing the threshold of 0.05, attesting to the dearth of significant heterogeneity within the instrumental variables of this inquiry. Notably, these findings underscore the imperviousness of the results regarding the influence of genetic pleiotropy.

### 3.4 Reverse MR analysis

In the realm of intestinal microbiota, specifically comprising two familial entities and four generative cohorts, whose causal affiliations with ACLD were prominently underscored through the lens of forward MR analysis, a subsequent deconstruction was embarked upon via reverse MR scrutiny. The findings thereof failed to unveil any discernible causal nexus between ACLD and the intricate tapestry of intestinal flora, as substantiated by the statistical insignificance denoted in Table 1 (*p* > 0.05). The exposure data from the reverse MR analysis are detailed in Supplementary Tables 3, 4.

**Table 1:** Results of reverse MR analysis.

Exposure	Outcome	SNP	Method	*β*	*SE*	*p*
PBC	Genus *Clostridium innocuum* group	10	IVW	0.0002	0.228	0.990
PBC	Genus Butyricicoccus	12	IVW	0.017	0.012	0.164
PBC	Genus Erysipelatoclostridium	12	IVW	0.007	0.014	0.5812
PSC	Genus *Eubacterium hallii* group	5	IVW	−0.008	0.026	0.7478
PSC	Family Clostridiaceae1	5	IVW	−0.029	0.0278	0.297
PSC	Family Lachnospiraceae	5	IVW	0.012	0.025	0.625

## 4 Discussion

In this study, we utilized GWAS-pooled data from MiBioGen and Finngen, subjected to analysis using TSMR. Our findings revealed a negative correlation between the risk of PBC and the relative abundance of three genera: *Clostridium innocuum* group, *Butyricicoccus*, and *Erysipelatoclostridium*. Conversely, the relative abundance of the genus *Eubacterium hallii* group exhibited a positive correlation with the risk of PSC. Furthermore, we observed a negative correlation between the development of PSC and the relative abundance of family *Clostridiaceae1* and family *Lachnospiraceae*. These results contribute to our understanding of the intricate relationships between specific microbial taxa and risks associated with PBC and PSC.

An increasing body of research underscores the role of intestinal flora in the development of ACLD through the gut-liver axis. This pathway involves microbial translocation, which is a pivotal feature of ACLD pathogenesis (Blesl and Stadlbauer, 2021). Bacteria and their metabolites, entering the circulation, are recognized by hepatic immune receptors, initiating intrahepatic inflammatory responses that contribute to the development and progression of ACLD (Tripathi et al., 2018). Moreover, structural alterations in the intestinal flora result in dysregulation of hepatic and intestinal bile acid circulation. This dysregulation precipitates cholestasis and accumulation of bile acid toxicity, ultimately inducing ACLD and progressive liver injury (Mousa et al., 2021).

A study conducted by Tedesco et al. revealed that translocation of *Lactobacillus gasseri* triggered the production of interleukin-17 (IL-17) by hepatic γδ T-cell receptor-positive (γδ TCR) cells in mice. This translocation is associated with hepatic inflammatory injury. Additionally, γδ TCR cells were isolated from the livers of patients with PSC, implying that intestinal flora may play a role in inducing cholestatic liver disease. Specifically, the mediation of γδ TCR cells to produce IL-17 has been identified as a potential mechanism contributing to the development of cholestatic liver disease (Tedesco et al., 2018). Observational studies have further supported the link between dysbiosis of gut flora and ACLD. For instance, Sabino et al. reported a higher proportion of *Enterococcus*, *Fusobacterium*, and *Lactobacillus* genera in the feces of 66 Belgian patients with PSC compared to controls (Sabino et al., 2016). Furukawa et al. (2020) also found relevant associations; they identified decreased bacterial diversity, a high abundance of *Lactobacillus*, and a low abundance of *Clostridiales* in the intestines of patients with PBC compared to the healthy population. Furthermore, in the ursodeoxycholic acid (UDCA)-treated nonresponder group, there was a significant decrease in the *Faecalibacterium* genus. This suggests that the presence of *Faecalibacterium* may influence the long-term prognosis of patients with PBC.

In alignment with prior investigations, our study identified significant associations using MR analysis. Specifically, the genus *Clostridium innocuum* exhibited a negative correlation with the risk of developing PBC. Conversely, the genus *Eubacterium hallii* and the family *Lachnospiraceae* were positively and negatively associated with the development of PSC, respectively. Earlier research has posited the absence of beneficial *Clostridiales* commensals in the intestines of PBC patients (Furukawa et al., 2020). Abe et al. (2018) reported a reduction in *Clostridium subcluster XIVa* among PBC patients compared to controls. Furthermore, patients with PBC with abnormal hepatic function exhibited decreased levels of *Clostridium cluster IV* and *Clostridium subcluster XIVa*, particularly in those with abnormal liver function. These findings suggest a potential protective role for *Clostridium* in PBC and indicate its possible influence on liver function. Scholars have affirmed that *Clostridium*, through 7-α-Dehydroxylation, converts Primary bile acids into secondary bile acids (Staley et al., 2017). Given that ACLD patients often experience bile acid metabolism dysregulation, reduced Dehydroxylation reaction, and cytotoxicity, the concentration of hydrophobic Primary bile acids increases, leading to toxicity accumulation and bile duct damage (Schmucker et al., 1990; Li et al., 2017; Torres et al., 2018; Fiorucci et al., 2021). Therefore, *Clostridium* may mitigate the risk of PBC development by improving bile acid metabolism. In a study by Kummen et al. in 2020, fecal DNA sequencing of patients with PSC revealed a significant depletion of various *Eubacterium* species (Kummen et al., 2021). Concurrently, patient treatment with UDCA was associated with elevated levels of *Eubacterium rectale*. While this study suggests a protective role for *Eubacterium* in PSC, our findings indicate that the genus *Eubacterium hallii* is a risk factor for PSC, necessitating further exploration of its role in PSC development. Quraishi et al. (2020) in the same year observed a notable reduction in the family *Lachnospiraceae* in patients with PSC, suggesting a potential protective effect against PSC. Previous research has confirmed the ability of family *Lachnospiraceae* to produce butyrate (Winston et al., 2021), a short-chain fatty acid known for its anti-inflammatory, bile acid metabolism-regulating, and intestinal barrier-improving properties (Cui et al., 2018; Ye et al., 2021; Liu et al., 2022; Wang et al., 2023). This may help mitigate inflammatory damage associated with bile acid stagnation and bacterial translocation. An earlier study in 2018 demonstrated a positive correlation between *Faecalibacterium* enrichment and *Erysipelatoclostridium* in healthy infants, which was not observed in cholestatic infants (Guo et al., 2018). This suggests a potential association between dysregulation of genus *Erysipelatoclostridium* and cholestasis. However, protective associations for PBC necessitate further investigation. While no direct study establishes the association of genus *Butyricicoccus* and family *Clostridiaceae1* with ACLD, but because genus *Butyricicoccus* is the main microbiota producing butyrate, it was found to be positively associated with the concentration of Secondary bile acids, which may contribute to the conversion of Primary bile acids to Secondary bile acids (Yang et al., 2021). Certain genera within family *Clostridiaceae*, such as *Clostridium*, have been implicated in the conversion of Primary bile acids to Secondary bile acids (Furukawa et al., 2020). Therefore, we posit that genus *Butyricicoccus* and family *Clostridiaceae1* may exert protective effects against ACLD through the production of butyrate and regulation of bile acids. Further research is warranted to validate these hypotheses.

It is also worth noting that the occurrence and development of non-alcoholic fatty liver disease (NAFLD) is also closely related to dysregulation of gut microbiota and bile acid metabolism, for example (Sorrentino et al., 2005). Qilong Zhai et al. found in an MR study that *Enterobacteriales*, *Enterobacteriaceae*, *Lachnospiraceae UCG-004*, and *Prevotella9* increased the risk of NAFLD, and *Dorea* and *Veillonella* increased the risk of NASH. *Oscillospira* and *Ruminococcaceae UCG-013* were able to reduce their risk, with *Veillonella*, *Lachnospiraceae UCG-004* being able to participate in bile acid metabolism. It has also been suggested that dysregulation of BA homeostasis and signaling may further contribute to abnormal lipid metabolism and lipotoxicity in NAFLD (Arab et al., 2017), and functional cholestasis may be related to the pathogenesis of NASH (Segovia-Miranda et al., 2019). Thus, it can be seen that the disorder of bile acid hepatic and intestinal circulation caused by dysbiosis of gut microbiota is likely to be an important cause of the development and progression of NAFLD, which has some overlap with the mechanism of ACLD triggered by gut microbiota, and thus it has been pointed out that cholestasis and NAFLD can share some of the therapeutic targets in their treatments (Trauner and Fuchs, 2022). Most of the positive gut microbiota we found in this study exerted their promotional or inhibitory effects on ACLD by affecting bile acid metabolism, and it should be noted that two protective genera, *Clostridium* and *Butyricicoccus*, were also found to be in increased abundance in patients with NAFLD regression after bariatric surgery in this study (Pérez-Rubio et al., 2023), and thus we hypothesize that they may be able to contribute to the development of common therapeutic targets to ameliorate both cholestasis and NAFLD by modulating the gut microbiota.

In comparison with previous literature, we found some similarities in the design of the two studies, but there are obvious differences in data sources, findings and research methods, etc. The study of Yang et al. (2024) found that order *Bacillales*, family *Peptostreptococcaceae*, family *Ruminococcaceae*, genus *Anaerotruncu* was associated with a reduced risk of developing PBC, order *Selenomonadales*, *family Bifidobacteriaceae* may be a factor that increases the risk of PBC, *order Selenomonadales*, *family Rhodospirillaceae*, and *genus RuminococcaceaeUCG013* was positively associated with PSC, *order Actinomycetales*, *family Actinomycetaceae*, *genus Actinomyces*, *genus Alloprevotella*, *genus Barnesiella*, and *genus Peptococcus* were negatively correlated with PSC risk. Zhang et al. (2023) similarly found that *Order Selenomonadales*, *Order Bifidobacteriales* and *Genus Lachnospiraceae UCG_004* were associated with higher risk of PBC, and *Family Peptostreptococcaceae* and *Family Ruminococcaceae* were protective against PBC. The above two studies and the content of the present study are from different databases, so the results produce a large difference, which also indicates that there may be some differences in the gut microorganisms affecting the pathogenesis of PBC and PSC in different sample populations. This study can further add candidate gut microorganisms affecting the pathogenesis of cholestatic liver disease, providing more complete basic research content for future studies.

The strengths and limitations of this study are delineated as follows. Notably, the utilization of the MR method stands out as a strength, enabling the analysis of the causal relationship between GM and ACLD, and can be complemented by the results of previous studies. This approach offers potential candidate gut microorganisms for subsequent investigations. Additionally, the study leveraged genetic data derived from a substantial population sample, mitigating the impact of confounding factors more effectively than small observational studies. Consequently, the results were deemed more reliable. However, certain limitations merit consideration. Firstly, the GWAS data primarily originated from European populations, raising the possibility of variation in results when extrapolated to other ethnic groups. Furthermore, the reliance on 16S rRNA gene sequencing limits taxonomic resolution to the genus level, precluding an exploration of the causal relationship between GM and ACLD at the species level. In specific instances, such as the prominence of the genus *Eubacterium hallii* in this study, discrepancies in its direction of action compared to prior research on its correlation with PSC were observed. Additionally, the absence of direct evidence confirming a causal relationship between the genus *Butyricicoccus* and the family *Clostridiaceae1* with ACLD pathogenesis is acknowledged. This underscores the necessity for future studies to validate and substantiate these potential associations. Consequently, the study calls for further investigation to solidify the understanding of these relationships and their implications for ACLD development.

In summary, this study identified six intestinal microbial taxa with causal associations to PBC and PSC through Mendelian randomization analysis. This finding establishes a foundational basis for investigating the link between GM and ACLD. Nevertheless, to comprehensively illustrate the specific mechanisms underlying the protective or triggering effects of these relevant flora on ACLD, further investigations employing large sample clinical Randomized Controlled Trial (RCT), pathway analyses, and laboratory studies are imperative.

## Data availability statement

The datasets presented in this study can be found in online repositories. The names of the repository/repositories and accession number(s) can be found in the article/Supplementary material.

## Ethics statement

No animal/human studies are presented in the manuscript.

## Author contributions

YC: Data curation, Methodology, Writing – original draft, Investigation. YG: Methodology, Software, Writing – original draft. YK: Data curation, Software, Writing – original draft. GZ: Supervision, Writing – review & editing, Project administration.

## References

[ref1] Abe K. Takahashi A. Fujita M. Imaizumi H. Hayashi M. Okai K.. (2018). Dysbiosis of oral microbiota and its association with salivary immunological biomarkers in autoimmune liver disease. PLoS One 13:e0198757. doi: 10.1371/journal.pone.0198757, PMID: 29969462 PMC6029758

[ref2] Arab J. P. Karpen S. J. Dawson P. A. Arrese M. Trauner M. (2017). Bile acids and nonalcoholic fatty liver disease: molecular insights and therapeutic perspectives. Hepatology 65, 350–362. doi: 10.1002/hep.28709. PMID: 27358174, PMID: 27358174 PMC5191969

[ref3] Blesl A. Stadlbauer V. (2021). The gut-liver axis in cholestatic liver diseases. Nutrients 13:1018. doi: 10.3390/nu13031018, PMID: 33801133 PMC8004151

[ref4] Burgess S. Thompson S. G. (2017). Interpreting findings from Mendelian randomization using the MR-egger method. Eur. J. Epidemiol. 32, 377–389. doi: 10.1007/s10654-017-0255-x, PMID: 28527048 PMC5506233

[ref5] Cui H. Cai Y. Wang L. Jia B. Li J. Zhao S.. (2018). Berberine regulates Treg/Th17 balance to treat ulcerative colitis through modulating the gut microbiota in the Colon. Front. Pharmacol. 9:571. doi: 10.3389/fphar.2018.00571, PMID: 29904348 PMC5991375

[ref6] Fiorucci S. Distrutti E. Carino A. Zampella A. Biagioli M. (2021). Bile acids and their receptors in metabolic disorders. Prog. Lipid Res. 82:101094. doi: 10.1016/j.plipres.2021.101094 33636214

[ref7] Fukui H. (2019). Role of gut dysbiosis in liver diseases: what have we learned so far? Diseases 7:58. doi: 10.3390/diseases7040058, PMID: 31726747 PMC6956030

[ref8] Furukawa M. Moriya K. Nakayama J. Inoue T. Momoda R. Kawaratani H.. (2020). Gut dysbiosis associated with clinical prognosis of patients with primary biliary cholangitis. Hepatol. Res. 50, 840–852. doi: 10.1111/hepr.13509, PMID: 32346970

[ref9] Greenland S. (2000). An introduction to instrumental variables for epidemiologists. Int. J. Epidemiol. 29, 722–729. doi: 10.1093/ije/29.4.722, PMID: 10922351

[ref10] Guo C. Li Y. Wang P. Li Y. Qiu C. Li M.. (2018). Alterations of gut microbiota in cholestatic infants and their correlation with hepatic function. Front. Microbiol. 9:2682. doi: 10.3389/fmicb.2018.02682, PMID: 30483228 PMC6243132

[ref11] Hemani G. Zheng J. Elsworth B. Wade K. H. Haberland V. Baird D.. (2018). The MR – base platformsupports systematic causal inference across the humanphenome. eLife 7:e34408. doi: 10.7554/eLife.34408, PMID: 29846171 PMC5976434

[ref12] Kummen M. Thingholm L. B. Rühlemann M. C. Holm K. Hansen S. H. Moitinho-Silva L.. (2021). Altered gut microbial metabolism of essential nutrients in primary sclerosing cholangitis. Gastroenterology 160, 1784–1798.e0. doi: 10.1053/j.gastro.2020.12.058, PMID: 33387530 PMC7611822

[ref13] Kurilshikov A. Medina-Gomez C. Bacigalupe R. Radjabzadeh D. Wang J. Demirkan A.. (2021). Large-scale association analyses identify host factors influencing human gut microbiome composition. Nat. Genet. 53, 156–165. doi: 10.1038/s41588-020-00763-1, PMID: 33462485 PMC8515199

[ref14] Kurki M. I. Karjalainen J. Palta P. Sipilä T. P. Kristiansson K. Donner K. M.. (2023). FinnGen provides genetic insights from a well-phenotyped isolated population. Nature 613, 508–518. doi: 10.1038/s41586-022-05473-8. PMID: 36653562, PMID: 36653562 PMC9849126

[ref15] Lammert C. Shin A. Xu H. Hemmerich C. O'Connell T. M. Chalasani N. (2021). Short-chain fatty acid and fecal microbiota profiles are linked to fibrosis in primary biliary cholangitis. FEMS Microbiol. Lett. 368:fnab038. doi: 10.1093/femsle/fnab038, PMID: 33836051 PMC8062329

[ref16] Li Y. Tang R. Leung P. S. C. Gershwin M. E. Ma X. (2017). Bile acids and intestinal microbiota in autoimmune cholestatic liver diseases. Autoimmun. Rev. 16, 885–896. doi: 10.1016/j.autrev.2017.07.002, PMID: 28698093

[ref17] Li P. Wang H. Guo L. Gou X. Chen G. Lin D.. (2022). Association between gut microbiota and preeclampsia-eclampsia: a two-sample Mendelian randomization study. BMC Med. 20:443. doi: 10.1186/s12916-022-02657-x, PMID: 36380372 PMC9667679

[ref18] Liu X. Wang L. Tan S. Chen Z. Wu B. Wu X. (2022). Therapeutic effects of Berberine on liver fibrosis are associated with lipid metabolism and intestinal flora. Front. Pharmacol. 13:814871. doi: 10.3389/fphar.2022.814871 35308208 PMC8924518

[ref19] Liwinski T. Heinemann M. Schramm C. (2022). The intestinal and biliary microbiome in autoimmune liver disease-current evidence and concepts. Semin. Immunopathol. 44, 485–507. doi: 10.1007/s00281-022-00936-6, PMID: 35536431 PMC9088151

[ref20] Mousa O. Y. Juran B. D. McCauley B. M. Vesterhus M. N. Folseraas T. Turgeon C. T.. (2021). Bile acid profiles in primary sclerosing cholangitis and their ability to predict hepatic decompensation. Hepatology 74, 281–295. doi: 10.1002/hep.31652, PMID: 33226645 PMC8141059

[ref21] Pérez-Rubio Á. Soluyanova P. Moro E. Quintás G. Rienda I. Periañez M. D.. (2023). Gut microbiota and plasma bile acids associated with non-alcoholic fatty liver disease resolution in bariatric surgery patients. Nutrients 15:3187. doi: 10.3390/nu15143187. PMID: 37513605, PMID: 37513605 PMC10385764

[ref22] Quraishi M. N. Acharjee A. Beggs A. D. Horniblow R. Tselepis C. Gkoutos G.. (2020). A pilot integrative analysis of colonic gene expression, gut microbiota, and immune infiltration in primary sclerosing cholangitis-inflammatory bowel disease: association of disease with bile acid pathways. J. Crohns Colitis 14, 935–947. doi: 10.1093/ecco-jcc/jjaa021, PMID: 32016358 PMC7392170

[ref23] Richardson N. Wootton G. E. Bozward A. G. Oo Y. H. (2022). Challenges and opportunities in achieving effective regulatory T cell therapy in autoimmune liver disease. Semin. Immunopathol. 44, 461–474. doi: 10.1007/s00281-022-00940-w, PMID: 35641679 PMC9256571

[ref24] Sabino J. Vieira-Silva S. Machiels K. Joossens M. Falony G. Ballet V.. (2016). Primary sclerosing cholangitis is characterised by intestinal dysbiosis independent from IBD. Gut 65, 1681–1689. doi: 10.1136/gutjnl-2015-311004, PMID: 27207975 PMC5036217

[ref25] Schmucker D. L. Ohta M. Kanai S. Sato Y. Kitani K. (1990). Hepatic injury induced by bile salts: correlation between biochemical and morphological events. Hepatology 12, 1216–1221. doi: 10.1002/hep.1840120523, PMID: 2227821

[ref26] Segovia-Miranda F. Morales-Navarrete H. Kücken M. Moser V. Seifert S. Repnik U.. (2019). Three-dimensional spatially resolved geometrical and functional models of human liver tissue reveal new aspects of NAFLD progression. Nat. Med. 25, 1885–1893. doi: 10.1038/s41591-019-0660-7, PMID: 31792455 PMC6899159

[ref27] Sorrentino P. Tarantino G. Perrella A. Micheli P. Perrella O. Conca P. (2005). A clinical-morphological study on cholestatic presentation of nonalcoholic fatty liver disease. Dig. Dis. Sci. 50, 1130–1135. doi: 10.1007/s10620-005-2719-1. PMID: 15986869, PMID: 15986869

[ref28] Staiger D. Stock J. H. (1997). Instrumental variables regression with weak instruments. Econometrica 65, 557–586. doi: 10.2307/2171753

[ref29] Staley C. Weingarden A. R. Khoruts A. Sadowsky M. J. (2017). Interaction of gut microbiota with bile acid metabolism and its influence on disease states. Appl. Microbiol. Biotechnol. 101, 47–64. doi: 10.1007/s00253-016-8006-6, PMID: 27888332 PMC5203956

[ref30] Storey J. D. Tibshirani R. (2003). Statistical significance for genomewide studies. Proc. Natl. Acad. Sci. U. S. A. 100, 9440–9445. doi: 10.1073/pnas.1530509100, PMID: 12883005 PMC170937

[ref31] Tedesco D. Thapa M. Chin C. Y. Ge Y. Gong M. Li J.. (2018). Alterations in intestinal microbiota lead to production of interleukin 17 by intrahepatic γδ T-cell receptor-positive cells and pathogenesis of cholestatic liver disease. Gastroenterology 154, 2178–2193. doi: 10.1053/j.gastro.2018.02.019, PMID: 29454797 PMC5985208

[ref32] Torres J. Palmela C. Brito H. Bao X. Ruiqi H. Moura-Santos P.. (2018). The gut microbiota, bile acids and their correlation in primary sclerosing cholangitis associated with inflammatory bowel disease. United European Gastroenterol J 6, 112–122. doi: 10.1177/2050640617708953, PMID: 29435321 PMC5802676

[ref33] Trauner M. Fuchs C. D. (2022). Novel therapeutic targets for cholestatic and fatty liver disease. Gut 71, 194–209. doi: 10.1136/gutjnl-2021-324305. PMID: 34615727, PMID: 34615727 PMC8666813

[ref34] Tripathi A. Debelius J. Brenner D. A. Karin M. Loomba R. Schnabl B.. (2018). The gut-liver axis and the intersection with the microbiome. Nat. Rev. Gastroenterol. Hepatol. 15, 397–411. doi: 10.1038/s41575-018-0011-z, PMID: 29748586 PMC6319369

[ref35] Verbanck M. Chen C. Y. Neale B. do R. (2018). Detection of widespread horizontal pleiotropy in causal relationships inferred from mendelian randomization between complex traits and diseases. Nat. Genet. 50, 693–698. doi: 10.1038/s41588-018-0099-7, PMID: 29686387 PMC6083837

[ref36] Wang S. Xiang L. li F. Deng W. lv P. Chen Y. (2023). Butyrate protects against *Clostridium difficile* infection by regulating bile acid metabolism. Microbiol. Spectr. 11:e0447922. doi: 10.1128/spectrum.04479-22, PMID: 37350595 PMC10434071

[ref37] Winston J. A. Rivera A. Cai J. Patterson A. D. Theriot C. M. (2021). Secondary bile acid ursodeoxycholic acid alters weight, the gut microbiota, and the bile acid pool in conventional mice. PLoS One 16:e0246161. doi: 10.1371/journal.pone.0246161, PMID: 33600468 PMC7891722

[ref38] Xie J. Huang H. Liu Z. Li Y. Yu C. Xu L.. (2023). The associations between modifiable risk factors and nonalcoholic fatty liver disease: a comprehensive mendelian randomization study. Hepatology 77, 949–964. doi: 10.1002/hep.32728, PMID: 35971878

[ref39] Xue H. Shen X. Pan W. (2021). Constrained maximum likelihood-based Mendelian randomization robust to both correlated and uncorrelated pleiotropic effects. Am. J. Hum. Genet. 108, 1251–1269. doi: 10.1016/j.ajhg.2021.05.014, PMID: 34214446 PMC8322939

[ref40] Yang Z. H. Liu F. Zhu X. R. Suo F. Y. Jia Z. J. Yao S. K. (2021). Altered profiles of fecal bile acids correlate with gut microbiota and inflammatory responses in patients with ulcerative colitis. World J. Gastroenterol. 27, 3609–3629. doi: 10.3748/wjg.v27.i24.3609, PMID: 34239273 PMC8240054

[ref41] Yang J. Ma G. Wang K. Yang H. Jiang S. Fan Q.. (2024). Causal associations between gut microbiota and Cholestatic liver diseases: a Mendelian randomization study. Front. Med. 11:1342119. doi: 10.3389/fmed.2024.1342119. PMID: 38327703, PMID: 38327703 PMC10847275

[ref42] Ye X. Shen S. Xu Z. Zhuang Q. Xu J. Wang J.. (2021). Sodium butyrate alleviates cholesterol gallstones by regulating bile acid metabolism. Eur. J. Pharmacol. 908:174341. doi: 10.1016/j.ejphar.2021.174341, PMID: 34273384

[ref43] Zhang J. Wu G. Tang Y. Liu H. Ge X. Peng R.. (2023). Causal associations between gut microbiota and primary biliary cholangitis: a bidirectional two-sample Mendelian randomization study. Front. Microbiol. 14:1273024. doi: 10.3389/fmicb.2023.1273024, PMID: 38033598 PMC10684913

[ref44] Zhang L. Zi L. Kuang T. Wang K. Qiu Z. Wu Z.. (2023). Investigating causal associations among gut microbiota, metabolites, and liver diseases: a Mendelian randomization study. Front. Endocrinol. 14:1159148. doi: 10.3389/fendo.2023.1159148, PMID: 37476494 PMC10354516

